# 

**Published:** 1901-02-16

**Authors:** 


### NOTICE TO CORRESPONDENTS.

All MSS., Letters, Books for Review, and other matters intended for the Editor should be addressed THE EDITOR, "THE HOSPITAL," THE HOSPITAL BUILDING, 28 & 29 SOUTHAMPTON STREET, STRAND, W.C.

The Editor cannot undertake to return rejected MS., even when accompanied by stamped directed envelope.

**Notice to Advertisers and Subscribers.**

All Advertisements, Orders for Copies of THE HOSPITAL, and Business Communications should be addressed to **THE MANAGER (Not to the Editor)**, THE HOSPITAL, 28 & 29 Southampton Street Strand, London, W.C.

The Cost of Subscription, post free, is as follows:—Per week, 2½d.; per quarter, 2s. 8d.; per half-year, 5s. 3d.; per annum, 10s. 6d. Foreign:—Per annum, 15s. 2d., payable, in all cases, in advance.

**NOTICE.—Vol. XXVIII. of "The Hospital" (April to September, 1900), in handsome cloth binding, is now on sale at the Office of this Journal, price, 7s. 6d. Cases for binding the Numbers contained in this Volume 1s. 6d. Index to Vol. XXVIII., 2½d., post free.**

**The Monthly Part for February is now ready. Price 6d., by post 8d.**

### SCRAPS AND GLEANINGS.

MR. E. S. HEYWOOD, who has been chairman of the Manchester Infirmary Board for the past 25 years, has retired from the office for personal reasons.

DURING the past 32 years since which the Cookridge Convalescent Hospital has been opened nearly 40,000 patients have been received. During the past year 1,275 persons had been admitted in the 43 weeks when the institution was open.

THE workpeople's donations to the Leicester Infirmary during 1900 amounted to £3,545. This was a decrease of £62 upon last year, but in view of the calls on the workmen for war funds, &c., this is considered by the chairman as most satisfactory.

SINCE the Pendleton Provident Dispensary was opened twenty years ago 17,370 persons have received benefit and 671,297 prescriptions have been dispensed. The receipts during the past year were £1,034, including £832 odd from members' subscriptions.

THE Trinity Hospital, Leicester, built five and a half centuries ago, is now in process of demolition. The foundations, being thoroughly sound, are to be used for the principal portion of the eastern part of the new hospital. The new building will be twice as large as the old hospital.

AT the annual meeting of the Darlington Hospital a resolution was agreed to that it is desirable to increase the accommodation at the hospital, and this meeting requests the committee to continue their efforts in this direction. The chairman showed that for 20 additional beds £6,000 would be needed, as well as an addition of £800 to the income.

THE total number of patients treated at the Plymouth Royal Eye Infirmary since the commencement of the charity amounts to 85,154. The cost of the new building, furnishing, &c., up to last October was upwards of £16,568, and it is calculated that the new buildings will necessitate an increased annual expenditure of £300, making the total yearly expenditure about £1,200.

### The Student of Medicine.

### APPOINTMENTS.

**Hedda Alström**, M.B., B.S.Durh., appointed Assistant Medical Officer to the Newcastle-upon-Tyne Union Workhouse. **G. M. Arkle**, L.S.A., appointed Medical Officer of the Belmont Road Workhouse of the West Derby Union. **T. W. Bartlett**, L.R.C.P., L.R.C.S.Edin., appointed District Medical Officer of the Risbridge and Saffron Walden Unions. **E. H. Birchall**, L.R.C.P., L.R.C.S.Edin., appointed Medical Officer for the Walton Workhouse of the West Derby Union. **W. J. Carmichael**, M.B., C.M.Edin., appointed Certifying Factory Surgeon for the Darlington District. **Crochley Clapham**, M.D., F.R.C.P.E., appointed a Consulting Physician to the Royal Hospital, Sheffield. **W. R. Edmond**, M.B.Edin., appointed District and Workhouse Medical Officer of the Totnes Union. **E. G. Fowler**, M.R.C.S., L.R.C.P., appointed one of the House Physicians to the Leeds General Infirmary. **R. Fielding-Ould**, M.A., M.D.Oxon., appointed Assistant Lecturer at the School of Tropical Medicine, Liverpool. **W. A. Helm**, M.B., Ch.B.Vict., appointed Assistant Medical Officer to the Parish of Birmingham Infirmary. **C. D. Ingle**, M.R.C.S., L.R.C.P.Lond., appointed District Medical Officer of the Shepton Mallet Union. **A. L. Jones**, M.R.C.S.Eng., D.P.H.Camb., appointed District Medical Officer to the Gower Union. **R. J. Leeper**, L.R.C.S.I., L.A.H.Dubl., appointed Certifying Factory Surgeon for the Market Bosworth District of Leicestershire. **T. Percy Legg**, M.B., F.R.C.S., appointed Assistant Surgeon, with charge of out-patients, at the Royal Free Hospital, Gray's Inn Road, W.C. **E. Sydney Littlejohn**, B.A., M.D., C.M., appointed Visiting Medical Officer to the Thomas Walker Convalescent Hospital, Concord, Sydney. **T. B. Marshall**, M.R.C.S., L.R.C.P.Lond., appointed District Medical Officer to the Maldon Union. **Gilbert E. Mould**, M.R.C.S.Eng., L.R.C.P.Lond., appointed Physician for Mental Diseases to the Sheffield Royal Hospital. **R. H. Norman**, M.D.Lond., B.S., M.R.C.S., L.R.C.P., apppointed Honorary Physician to the Holloway and North Islington Dispensary. **Harold Douglas Singer**, M.D.Lond., M.R.C.S., L.R.C.P., appointed Junior House-Physician to the National Hospital for the Paralysed and Epileptic, Queen Square. **W. J. Spence**, L.R.C.P., L.R.C.S.Edin., appointed Certifying Factory Surgeon for the Syston District of Leicestershire. **M. B. Steuart**, M.B., C.M.Edin., appointed District Medical Officer to the Newton Abbott Union. **Andrew Stewart**, M.B., M.S.Glasg., appointed Visiting Medical Officer to the Jubilee Sanatorium for Consumption at Dalby, Queensland. **A. J. Troughton**, L.R.C.S., L.R.C.P.Edin., L.F.P.&S.Glasg., appointed Medical Officer of the Bentham District of the Settle Union. **A. H. Tubby**, M.S., F.R.C.S., appointed a Consulting Surgeon to the Vine Hip Hospital, Sevenoaks. **E. B. Waggett**, M.B., B.C.Camb., appointed Surgeon to the Out-patient Throat and Ear Department, Great Northern Central Hospital. **A. I. Webster**, M.B., C.M.Edin., appointed District Medical Officer of the Parish of Willesden.

